# Few dental indices in modern Bulgarian population from southern Bulgaria

**DOI:** 10.1186/s40101-023-00332-5

**Published:** 2023-07-22

**Authors:** Zdravka Harizanova, Atanas Baltadjiev, Ferihan Popova, Marieta Peycheva

**Affiliations:** 1 Department of Anatomy, Histology and Embryology, Faculty of Medicine, Medical University of Plovdiv, 15A Vassil Aprilov Blvd., Plovdiv, 4002 Bulgaria; 2 Department of Neurology, Faculty of Medicine, Medical University of Plovdiv, 15A Vassil Aprilov Blvd., Plovdiv, 4002 Bulgaria

**Keywords:** Physiological anthropology, Odontometry, Bulgarian population, Evolution, Dental reduction

## Abstract

**Aim:**

The aim of the present study is to evaluate the reduction of the distal teeth towards the medial ones in one functional dental group in southern Bulgarian population.

**Materials and methods:**

The study included 232 Bulgarians aged 20–40 years. Mesiodistal dimensions of the teeth were measured by Dentistry Sliding Vernier Caliper and analyzed with SPSS 23.0. Four interdental indices were calculated: inter-incisive, premolar, upper, and lower molar indices.

**Results:**

We found a decrease in the percentage ratio of the lateral to the central incisors of people from the Bulgarian population compared to those dating from the Eneolithic period on the territory of Bulgaria. Furthermore, we found a reduction in the percentage ratio of the upper and lower second molars compared to the first ones. The biggest reduction in the percentage ratio (more than 6%) was found in the lower second premolars compared to the first ones, which is characteristic for southern Bulgarians.

**Conclusion:**

There was a dental reduction in all the distal members compared to the medial ones participating in one morphological dental group. As a result, we think that interdental indices can be used for explaining historical, cultural, and biological macro and microevolutionary processes and thus for understanding the origin, formation, contacts, and migration pathways of the different populations leading to ethnic variation of humanity. Therefore, they can be a reliable source of information in physiological anthropology.

## Introduction

In anthropology, the ratios, between dimensions of the teeth from one morphological and functional group, are of great interest because they can assist in studying evolutionary trends in anthropological processes. The human masticatory system, which consists of superior jaw, inferior jaw, teeth, temporomandibular joint, and masticatory muscles, is functionally related not only to nutrition but also to articulated speech. For this reason, it has undergone enormous development in the history of mankind. The factors that led to this are environmental factors: change in the type of food, its preparation, use of fire, and different tools and change in climate and lifestyle, as well as genetic factors associated with various mutations, in relation to the upright posture, the increase in brain size, and the formation of language and speech.

There are many theories that are based on the change in the diet as the main factor in the reduction of the masticatory complex in humans [12, 15, 20]. With the discovery of the fact that the development of dental germs and their differentiation into different functional groups (incisors, canines, premolars, molars) is under the control of signal molecules that are stimulated by different genes, two theories are formed:

1. “Space theory” was proposed by Butler [5] and developed by Dahlberg [7]. Butler believed that in each dental arch, there is a concentration of morphological genes, the result of which is that each tooth resembles its neighbor. This concentration is highest when the first tooth of each morphological group is developing, so the medial tooth of each group is the most stable and is termed the key tooth. At the same time, the difference in it leads to variations in tooth morphology. With moving away from the key tooth, this concentration decreases for the more distally positioned tooth. As an inference to this theory, Dahlberg [7] believed that more variations are shown in the size of the distal teeth of each group, i.e., the variations in lateral incisors are greater than these in medial ones, the second premolars are greater than the first, and in the second upper and lower molars, they are larger than the first.

van Valen [21] developed Butler’s theory by suggesting that variation in tooth size within a group is due to a pre-pattern that is responsible for the development of tooth germs, and that evolves along different gradients leading to variation in distal teeth.

2. The second theory — “clone theory,” was proposed by Osborn [16]. According to this theory, there are three different patterns: for incisors, for canines, and for molars that form the human dentition. Development starts from these patterns, and then different gradients start to act, which explains the different variations in the same group. As progressing to the more distal tooth, the primary pattern changes according to the remaining available space.

Some authors such as Schwartz and Tattersall [18] believe that the reasons for the reduction of the dental dimensions of the distal teeth may be in the formation of the human chin as a result of the shortening of the lower jaw and the dental arch.

Other researchers such as Macho and Berner [13] believe that the reduction in the size of the second molars may be due to the formation of a helicoidal occlusal plane as well as the curve of Spee, which are considered to be a functional adaptation to the masticatory function unique to humans. An important factor according to Osborn [17] is also the evolution in the position of the temporomandibular joint as well as the mandibular ramus. In modern man, these anatomical structures move forward, which is important for the occlusion of the upper and lower molars in mastication, the length of the lower jaw is shortened.

Cziko [6] points out that the evolution of the maxillofacial system is also related to the development of the brain, which occurs as a result of verbal communication between individuals. According to Aiello and Dean [2], another reason for that is the upright posture, since the smaller size of the oral cavity supports the center of gravity of the human skull and therefore facilitates balance. Stedman et al. [19] also support this theory, stating that the encephalization of the human species lead to a gracilization of the maxillofacial region and hence a reduction in tooth sizes.

*The aim* of this study was to determine certain values of some interdental indices in South Bulgarians and to assess the degree of reduction of the distal tooth towards the medial one in one morphological dental group.

## Materials and methods

The study samples consisted of 232 individuals (121 males and 111 females) of Bulgarian origin living in southern Bulgaria aged 20–40 years. Patients were included based on the following criteria: Bulgarian ethnicity, the presence of complete set of fully erupted and periodontally healthy teeth, the presence of non-carious and non-worn teeth, no dental history of any crown restorations or bridges, and normal occlusion. Patients with orthognathic surgery or trauma, history or clinical evidence of cleft palate, history or clinical features suggestive of endocranial disorders, metabolic disorders, developmental disorders, and history of prolonged illness were excluded.

Mesiodistal measurements of the teeth were obtained by Dentistry Sliding Vernier Caliper, Ridge Mapping Caliper type A and type B. We used the method of direct anthropological technique, modified by Prof. Yordanov et al. [22]. The maximum mesiodistal dimension was assessed as being the maximum distance between the mesial and distal proximal surfaces of the tooth crown, usually in the upper or middle third of coronal height. It is also termed the dental width.

We calculated the following interdental indices:

- *Inter-incisive index* as ratio of the mesiodistal dimensions of maxillary lateral incisor to the mesiodistal dimensions of the maxillary central incisor (MD12/MD11) × 100
- *Index of the premolars* as ratio of the mesiodistal dimensions of second lower premolar to the same of the first one, *PI* = (MD45/MD44) × 100
- *Index of the upper molars* as ratio of the mesiodistal dimension of the second upper molar to the same of the first one, *UMI* = (MD17/MD16) × 100
- *Index of lower molars* as ratio of the mesiodistal dimension of the second lower molar to the same of the first one, *LMI* = (MD47/MD46) × 100

The measurements were analyzed with SPSS 23.0 using Student’s *t*-test. The level of statistical significance was set at *p* < 0.05. The degree of significance was considered weak (*p* < 0.05), moderate (0.01 > *p* > 0.001), or high (*p* < 0.001).

An ethical approval was taken for this study by the ethics committee in Medical University-Plovdiv. Informed consents were taken from all patients involved in the study. All methods were performed in accordance with the relevant guidelines and regulations.

## Results

1. We found statistically significant difference with high degree of significance (*p* < 0.001) between mesiodistal dimensions of central incisors in South Bulgarian population and remnants from the Eneolithic period. Furthermore, we detected statistically significant difference with moderate degree of significance (*p* < 0.01) in mesiodistal dimensions of lateral incisors between the two populations. The inter-incisive index in the teeth found on the territory of Bulgaria dating from the Eneolithic period is 81.25%. The same in people from southern Bulgarian population studied in the current research is 80.44%. There is a decrease in this index, which shows a tendency in reducing the percentage ratio of the lateral upper incisor to the central one, without statistical significance (*p* > 0.05) (Table 1).
2. There was no statistically significant difference between mesiodistal dimensions of first lower premolars, but we found such in second lower premolars (*p* < 0.001). The index of premolars found on the territory of Bulgaria dating from the Eneolithic period is 104.62%. The same in people from southern Bulgarian population studied in the current research is 98.06%. The decrease in this index is statistically significant (*p* < 0.001), which shows a strong reduction of the percentage ratio of the second lower premolar compared to the first one (Table 2).
3. We also found statistically significant differences between mesiodistal dimension of upper first and second molars (*p* < 0.001). The index of the upper molars found on the territory of Bulgaria dating from the Eneolithic period is 96.88%. The same in people from southern Bulgarian population studied in the current research is 95.94%. There is a decrease in this index, which shows a tendency in reducing the percentage ratio of the second upper molar compared to the first one, without statistical significance (*p* > 0.05) (Table 3).
4. We did not find statistically significant difference between mesiodistal dimensions of inferior first and second molars (*p* > 0.05). The index of the lower molars, found on the territory of Bulgaria dating from the Eneolithic period, is 96.19%. The same in people from southern Bulgarian population studied in the current research is 95.22%. There is a decrease in this index, which shows a tendency in reducing the percentage ratio of the second lower molar compared to the first one, without statistical significance (*p* > 0.05) (Table 4).

**Table 1:** Comparison of the inter-incisor index in teeth found on Bulgarian territory during the Eneolithic period and in modern southern Bulgarian population

Parameters	Eneolithic period				Current study					*P*
	*N*	Mean	*SD*	*SE*	Parameters	*N*	Mean	*SD*	*SE*	
I11MD	98	8.0	0.65	0.07	I11MD	232	8.44	0.66	0.11	** < 0.001**
I12MD	98	6.5	0.75	0.08	I12MD	232	6.76	0.71	0.11	** < 0.01**
Incisive index	98	81.25	8.20	0.83	Incisive index	232	80.44	8.44	0.91	> 0.05

**Table 2:** Comparison of the index of premolars found on Bulgarian territory during the Eneolithic period and in modern southern Bulgarian population

Parameters	Eneolithic period				Current study					*P*
	*N*	Mean	*SD*	*SE*	Parameters	*N*	Mean	*SD*	*SE*	
I44MD	98	6.5	0.64	0.07	I44MD	232	6.46	0.68	0.11	> 0.05
I45MD	98	6.8	0.71	0.07	I45MD	232	6.33	0.78	0.13	**< 0.001**
Premolar index	98	104.62	6.59	0.67	Premolar index	232	98.06	6.78	0.73	**< 0.001**

**Table 3:** Comparison of the index of the upper molars, found on Bulgarian territory during the Eneolithic period and in modern southern Bulgarian population

Parameters	Eneolithic period				Current study					*P*
	*N*	Mean	*SD*	*SE*	Parameters	*N*	Mean	*SD*	*SE*	
I16MD	98	9.60	0.70	0.07	I16MD	232	10.32	0.74	0.10	**< 0.001**
I17MD	98	9.30	1.09	0.11	I17MD	232	9.77	1.13	0.08	**< 0.001**
Upper molar index	98	96.88	5.19	0.52	Upper molar index	232	95.94	5.42	0.58	> 0.05

**Table 4:** Comparison of the index of the lower molars, found on Bulgarian territory during the Eneolithic period and in modern southern Bulgarian population

Parameters	Eneolithic period				Current study					*P*
	*N*	Mean	*SD*	*SE*	Parameters	*N*	Mean	*SD*	*SE*	
I46MD	98	10.50	0.72	0.07	I46MD	232	10.56	0.70	0.12	> 0.05
I47MD	98	10.10	0.51	0.05	I47MD	232	10.03	0.50	0.08	> 0.05
Lower molar index	98	96.19	5.20	0.53	Lower molar index	232	95.22	5.13	0.56	> 0.05

## Discussion

Eneolithic period is a transitional period between the Neolithic period and the Bronze Age, during which the earliest metallic (copper) artifacts appeared; hence, it is also known as the Copper Age (4800–2000 BC). Artifacts from this period have been found throughout the Balkan Peninsula. This is the historical period when agricultural tribes began their migration from Western Asia to Europe. The Balkan Peninsula became the most important route for resettlement. After them, many different tribes settled on the territory of modern Bulgaria, such as Thracians, Greeks, Roman colonists, Slavs, proto-Bulgarians, and Turks. Some of them belong to the Indo-European language group, but others belong to the Turkish language group. The mentioned populations have been successfully assimilated. The consequences were a very large genetic diversification of the modern Bulgarian population. Regardless of the genetic diversity, it should be noted that all the listed people belong to the species Homo sapiens. The situation is similar throughout the European continent. There are no pure genetically ethnicities. In order to achieve greater scientific precision, we excluded patients with non-Bulgarian origins from the study.

Information about the Eneolithic population was taken from the studies of Prof. Yordanov [22], who researched and described odontometric dimensions of archeological discoveries (skulls) found on the territory of Bulgaria.

In our study, we calculate the interdental indices of objects from southern Bulgarian population: the inter-incisive index, which shows the percentage of the upper lateral incisor to the upper central one; the premolar index, which shows the percentage of the lower second premolar to the lower first premolar; and the step indices of the molars, which show the percentage of the upper and lower second molars to the respective upper and lower first molars. Then, we calculate these indices for the teeth found on the territory of Bulgaria from the Eneolithic period [22]. When comparing them, we found a decrease in the values of these indices in modern man, which confirms the theories of reducing dental size. According to our study, the size of the distal members of each morphological tooth group (incisors, premolars, and molars) is reduced compared to that of their medial neighbors. The greatest reduction is in the percentage of the second lower premolar to the first. We detected a tendency in the reduction of the percentage of the lateral upper incisor to the central upper incisor. These findings are confirmed by Abadjiev [1]. A possible reason for this is the simpler morphology of the lateral upper incisor, which makes it more susceptible to mutations and other factors. Reduction of the inter-incisive index, i.e., reduction of the percentage of the lateral incisor to the central one, has been reported by other researchers such as Brook et al. [4] and Gungor and Turkkahraman [9]. Endo et al. [8], who studied these ratios in the Japanese, confirmed that the percentage of the lateral upper incisor with the central one exhibited the greatest reduction. In our study, however, the largest reduction is in the percentage of the lower second premolar to the first. Similar results were obtained by Norihisa et al. [14]. Furthermore, we find a tendency of reduction in the indices of the upper and lower molars, which shows a decrease in the percentage of the second upper and lower molars compared to the first upper and lower molars, respectively. We believe the main reason is the population migration as well as human evolution. For example, canines in prehistoric man are longer than other teeth, and the second molar is the largest tooth in human mouth. In modern man, this has changed as the size of the canines has decreased, and their level has reached the occlusal surface of other teeth. The mesiodistal dimension of the second upper molars also decreases significantly, so that in modern man the first upper molar has the largest mesiodistal dimension. These processes have been described by Haile-Selassie [10], as well as by Brace et al. [3]. Some authors, such as Schwartz and Tattersall [18], believe that the reasons for this may be the formation of the human chin, which is formed as a result of shortening of the lower jaw and dental arch. This contradicts the research of Ichim et al. [11], according to which the formation of the chin may be due to the reduction of perioral muscles, as well as that of the tongue.

## Conclusion

The biggest reduction with statistically significant difference in the percentage ratio (more than 6%) was found in the lower second premolars compared to the first ones, which is characteristic for southern Bulgarians.

We found a tendency in reducing the percentage ratio of the lateral towards the central incisors of people from southern Bulgarian population compared to those dating from the Eneolithic period on the territory of Bulgaria. We also detected a tendency in reducing the percentage ratio in the upper and lower second molars compared to the first ones.

As a result, we believe that interdental indices can be used for explaining historical, cultural, and biological macro and microevolutionary processes and thus for understanding the origin, formation, contacts, and migration pathways of the different populations leading to ethnic variation of humanity. Therefore, they can be reliable source of information in physiological anthropology.

## Data Availability

The paper is containing original research and has not been submitted/published earlier in any journal and is not being considered for publication elsewhere.
